# Ferroptosis contributes to quercetin-induced anti-cancer activity through blockade of LGR4/NF-κB/GPX4 axis in oral squamous cell carcinoma

**DOI:** 10.3389/fonc.2026.1835555

**Published:** 2026-07-14

**Authors:** Xiao-jiao Wang, Peng Zhang, Yue Gao, Yu Shao, Ling Chen

**Affiliations:** 1 Department of Pharmacy, Wuhan Third Hospital (Tongren Hospital of Wuhan University), Wuhan, Hubei, China; 2 Department of Pharmacy, Hubei Provincial Hospital of Traditional Chinese Medicine, Wuhan, Hubei, China; 3 Department of Pharmacy, Affiliated Hospital of Hubei University of Chinese Medicine, Wuhan, Hubei, China; 4 Institute of Traditional Chinese Medicine, Hubei Province Academy of Traditional Chinese Medicine, Wuhan, Hubei, China; 5 Department of Pharmacy, Changping Hospital of Traditional Chinese medicine, Beijing, China

**Keywords:** ferroptosis, LGR4, NF-κB, oral squamous cell carcinoma, quercetin

## Abstract

**Objective:**

Oral squamous cell carcinoma (OSCC) is a malignancy that faces challenges such as chemotherapy resistance and side effects. There is an urgent need for effective, low-toxicity compounds to treat OSCC. Here, we examined whether quercetin induces ferroptosis in OSCC cells and explored the potential molecular mechanisms.

**Methods:**

The role of LGR4/NF-κB/GPX4 in OSCC cells (CAL27 and SCC9) was studied through gene overexpression or RNA interference. Additionally, OSCC cell lines were treated with quercetin to examine its effects and underlying mechanisms in OSCC.

**Results:**

Quercetin dose-dependently reduced the viability of OSCC cells, while co-treatment with the ferroptosis inhibitor liproxstatin-1 significantly counteracted quercetin-induced cell death. RNA-seq analysis showed that quercetin’s inhibitory effect on OSCC cells is linked to ferroptosis induction. Quercetin concentration-dependently decreased GPX4 expression in OSCC cells by suppressing the LGR4/NF-κB signaling pathway. LGR4-induced ferroptosis inhibition was counteracted by either quercetin or an NF-κB inhibitor. Mechanistically, LGR4 could induce upregulation of IKKβ, leading to IκBα ubiquitination and degradation, which promotes NF-κB activation and GPX4 transcription, ultimately inhibiting ferroptosis in OSCC cells.

**Conclusions:**

Our findings indicate that ferroptosis may play a role in quercetin’s anti-OSCC activity by blocking the LGR4/NF-κB/GPX4 axis, which supports the potential use of quercetin as a therapeutic agent for OSCC.

## 1 Introduction

Oral squamous cell carcinoma (OSCC) is the most common malignancy of the oral cavity. The contributing factors for OSCC include the use of tobacco products in smoked or smokeless forms, alcohol, and human papillomavirus (1). The prognosis for OSCC remains poor due to rapid growth patterns and a high risk of metastasis and recurrence (2). Currently, surgical resection combined with chemotherapy or radiation therapy is the most common treatment strategy for OSCC, but the overall 5-year survival rate has not exceeded 50% (3). Additionally, most chemotherapeutic agents may lead to resistance and severe toxic side effects (4). Therefore, there is an urgent need to better understand OSCC and to develop safer, more effective anticancer therapy agents.

Leucine-rich repeat-containing G protein-coupled receptor 4 (LGR4), also known as GPR48, has been reported to be highly expressed in various cancers, such as OSCC (5). A recent study revealed that LGR4 deletion enhances ferroptosis when combined with chemotherapeutic drugs in colorectal cancer by downregulating the Wnt signaling pathway (6). Nuclear factor kappa-B (NF-κB) expression levels consistently contribute to cancer development and poorer prognosis (7). NF-κB regulates multiple functions of cancer stem cells, including stemness, metabolism, immunomodulatory activity, dormancy, and resistance to therapy (8). LGR4 has been shown to modulate the level and activity of NF-κB (9). A published study indicated that the LGR4/NF-κB cascade influences epithelial stem cell behavior and inflammatory responses to gland-invading Helicobacter pylori (10). Additionally, previous research has revealed that LGR4 activates the NF-κB signaling pathway and migration-related adhesion molecules, thereby promoting multiple myeloma cell homing (9). However, there is no clear evidence explaining how LGR4 affects cell death in OSCC and regulates the activation of NF-κB.

Quercetin (Qu), a natural polyphenolic compound, has many beneficial properties and is found to inhibit the development of cancers, including OSCC (11). Recent studies have shown that quercetin exerts anticancer effects by inducing ferroptosis and influencing specific molecular pathways in OSCC, such as NF-κB signaling (12). However, the exact roles and mechanisms of quercetin in OSCC remain unclear.

In this study, we conducted *in vitro* experiments to determine if quercetin can protect against OSCC by inducing ferroptosis through the inhibition of the LGR4/NF-κB pathway. Therefore, our findings provide a new approach for comprehensive treatment of OSCC.

## 2 Materials and methods

### 2.1 Chemical compounds and cell lines

Quercetin (Q4951), liproxstatin-1 (SML1414), helenalin (#374000), and RSL-3 (#SML2234) were purchased from Sigma-Aldrich (USA). CAL27, SCC9, and HOEC cells were obtained from the American Type Culture Collection (ATCC). All cell lines were cultured in Dulbecco’s Modified Eagle Medium (DMEM, Gibco, Invitrogen, USA). The DMEM media was supplemented with 10% fetal bovine serum (FBS, Gibco, Invitrogen, USA) and 1% Penicillin-Streptomycin (Sigma-Aldrich, USA). Cells were incubated in a humidified 5% CO2 incubator at 37 °C.

### 2.2 Cell counting kit 8

Cell viability was assessed using the CCK-8 assay. Briefly, 5 × 10^3 cells per well were cultured in a 96-well plate and incubated overnight before transfection or treatment. Next, 100 μL of medium containing 10 μL of CCK-8 solution was added, and the plates were incubated at 37 °C for 1.5 hours. Absorbance was measured at 450 nm using a microplate reader (Bio-Rad, Hercules, CA, USA).

### 2.3 Reactive oxygen species detection

ROS concentrations were measured using a cellular ROS assay kit (#Ab113851, Abcam, UK). Briefly, OSCC cells were cultured in 60 mm dishes. After treatment and transfection, cells were collected and resuspended in 500 μL of serum-free medium with 10 mM Dichloro-dihydro-fluorescein diacetate (DCFH-DA), then incubated for 30 minutes at 37 °C. The cells were then washed twice and resuspended in 300 μL phosphate-buffered saline (PBS). Fluorescence levels were analyzed via flow cytometry (Leica, Germany).

### 2.4 Glutathione (GSH) measurement

GSH levels were measured using an intracellular GSH detection assay kit (#A006-2-1, Nanjing Jiancheng, China). After treatment and transfection, OSCC cells were harvested and sonicated according to the manufacturer’s instructions. The supernatant was incubated with dithiodinitrobenzoic acid (DTNB). GSH concentrations were determined at 405 nm using flow cytometry (Leica, Germany).

### 2.5 Malondialdehyde measurement

MDA levels were determined using a lipid peroxidation (MDA) assay kit (#ab233471, Abcam, UK). OSCC cell lines were cultured in 10 cm dishes. Following transfection and treatment, the cells were harvested, homogenized, lysed, and centrifuged to collect supernatants. Thiobarbituric acid (TBA) was added to the supernatant, and absorbance was measured at 532 nm with a microplate reader (Bio-Rad, Hercules, CA, USA).

### 2.6 RNA high-throughput analysis

Following quercetin treatment of CAL27 cells, RNA was isolated using TRIzol reagent (ThermoFisher Scientific, USA). The processes of library preparation and RNA sequencing were then carried out completed.

### 2.7 Cell transfection

OSCC cells (1 × 10^5 cells per well) were cultured in a 6-well plate and incubated overnight. Once cell fusion reached 60%, the cells were transfected with the indicated plasmids using Lipofectamine 2000 (#11668-027, Invitrogen, USA) according to the manufacturer’s instructions. After 12 hours of incubation, the culture medium was replaced, and the cells were incubated for an additional 16 hours before analysis.

### 2.8 RNA extraction and quantitative PCR

After the indicated treatment or transfection, total RNA was isolated from the OSCC cell lines using TRIzol reagent (Invitrogen, USA) and then reverse-transcribed into cDNA using the PrimeScript™ RT reagent kit (Takara, Japan). To measure the expression of GPX4, qPCR was performed using the QuantiTect™ SYBR Green PCR Kit (Takara, Japan) with specific primers. The β-actin gene served as an internal control. Relative GPX4 mRNA expression was calculated using the 2−ΔΔCT method. The sequences of the specific primers are as follows:

GPX4 forward, 5’-CTGACCTTTATGATGATGCAGC-3’,

reverse, 5’-CAGGCTTTTCTTAGTTGATGCA-3’;

β-actin forward, 5’- CATGTACGTTGCTATCCAGGC-3’,

reverse, 5’- CTCCTTAATGTCACGCACGAT-3’.

### 2.9 Western blotting analysis

The total protein in OSCC cell lines was extracted using RIPA buffer (Thermo Scientific, USA) on ice for 30 minutes. The bicinchoninic acid (BCA) protein assay kit (Beyotime, China) was employed to determine the protein concentrations in each sample. Subsequently, equal amounts of protein were separated by 10% SDS-PAGE, transferred onto PVDF membranes (Merck Millipore, USA), and blocked with 5% non-fat milk for 1 hour. The membranes were incubated overnight at 4 °C with primary antibodies, including: NRF2 (ab62352), HO-1 (ab305290), GPX4 (ab125066), FTH1 (ab75973), TFR1 (ab109259), LGR4 (ab140845), NF-κB (ab283716), p-NF-κB (ab247871), IκBα (ab183503), IKKα (ab32041), IKKβ (ab124957), and β-actin (ab7817). On the following day, the membranes were incubated at room temperature for 1 hour with HRP-conjugated secondary antibodies. Finally, the protein bands were visualized using chemiluminescence kits and detected with chemiluminescence (Bio-Rad, CA, USA).

### 2.10 Luciferase reporter assay

The promoter region of GPX4 was amplified by PCR and cloned into the PGL3-basic vector. OSCC cell lines were cultured in 24-well plates for one day. Then, the cells were transfected with PGL-3GPX4/promoter and Renilla plasmids. After 24 hours, the cells were transfected again with or without NF-κB plasmid for 48 hours. Next, the cells were treated with or without quercetin for 24 hours. Firefly and Renilla luciferase activities were measured using a Dual-Luciferase Reporter Assay Kit (#RG027, Beyotime, China). The primer sequences used are as follows: GPX4 promoter-F: 5’-ATTTCTCTATCGATAGGTACC-3’; GPX4 promoter-R: 5’-CAGTACCGGAATGCCAAGCTT-3’.

### 2.11 Statistical analysis

Variables were presented as the mean ± S.D. Statistical analyses were performed using SPSS 20.0 (IBM, Chicago, IL, USA). Differences between the two groups were assessed with the unpaired two-tailed Student’s t-test. Comparisons among more than two groups were performed using one-way analysis of variance (ANOVA). A P value of less than 0.05 was considered statistically significant.

## 3 Results

### 3.1 Ferroptosis is correlated with quercetin-induced suppression in OSCC cells

**Figure 1:**
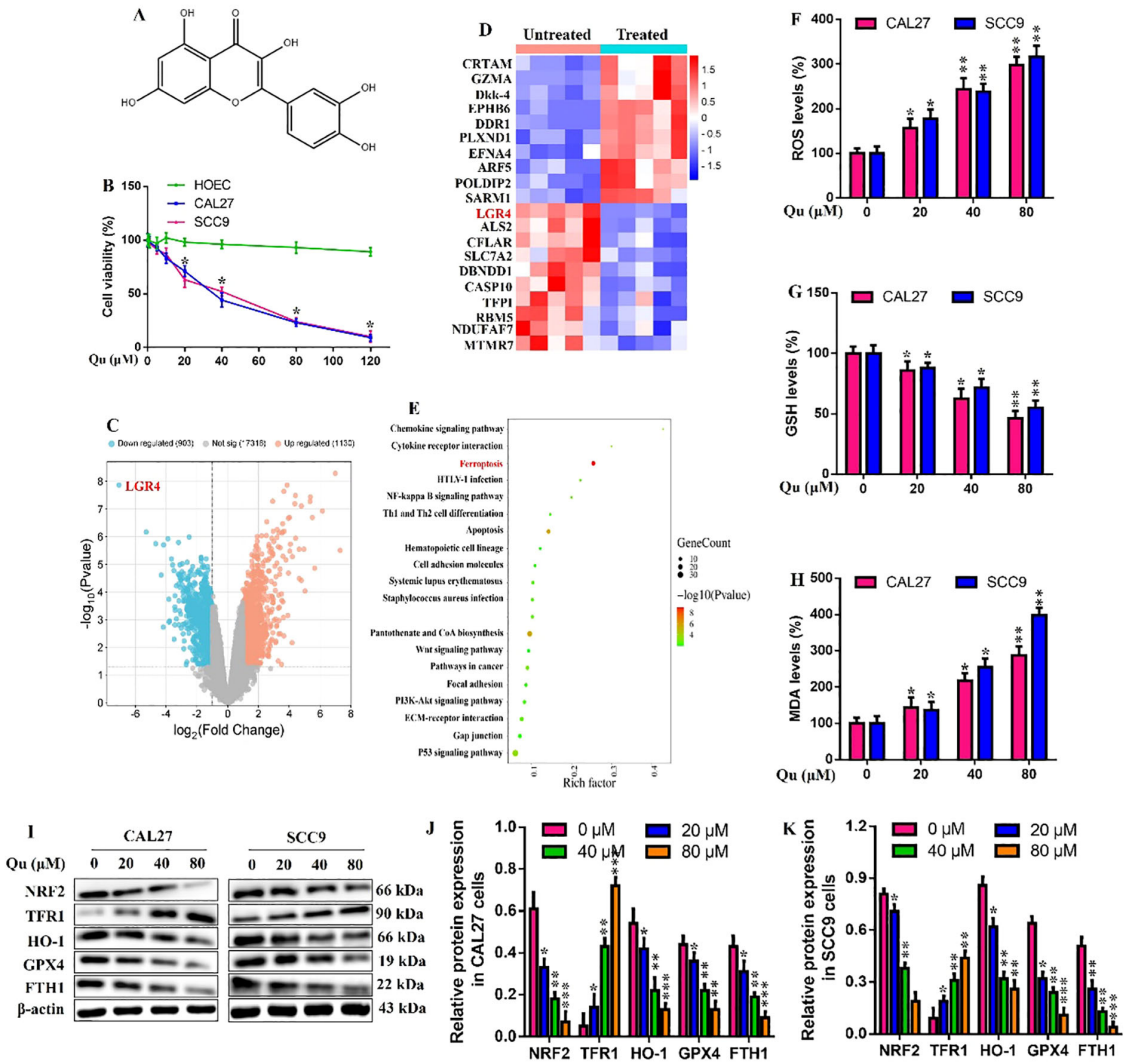
Ferroptosis induction is correlated with anti-OSCC effect of quercetin. **(A)** Chemical structure of quercetin (Qu). **(B)** Cell viability of CAL27, SCC9, and HOEC cell lines treated with quercetin was determined by the CCK-8 assay. **(C)** Volcano plot and **(D)** clustering heatmap for CAL27 cells treated with quercetin (40 μM). **(E)** KEGG pathway enrichment analysis was performed in CAL27 cells treated with quercetin (40 μM). Following treatment with quercetin for 24 h, **(F)** ROS, **(G)** GSH, **(H)** MDA, and **(I–K)** ferroptosis-associated proteins in CAL27 and SCC9 cells were determined. Data were shown as the mean ± SD; the number of biological replicates was 3, ^*^P < 0.05, ^**^P < 0.01, ^***^P < 0.001, compared with HOEG cells or 0 μM quercetin.

Consistent with previous reports (11, 12), our results demonstrated that quercetin decreases cell viability in OSCC cell lines as shown by the CCK-8 assay, while exhibiting lower toxicity in normal human oral epithelial cells (HOEC) (**Figure 1B**); the IC50 value of quercetin was approximate 40 μM, thus this concentration was used in subsequent experiments. To explore the potential mechanism behind quercetin’s anti-cancer effects, we conducted RNA-seq analysis comparing quercetin-treated cells with controls. **Figures 1C, D** showed that LGR4 was significantly reduced in cells treated with quercetin. KEGG analysis indicated that ferroptosis is strongly linked to the cell death caused by quercetin. (**Figure 1E**). Taken together, the above results suggest that quercetin triggers ferroptosis in OSCC cells. To further verify our conclusion, we examined characteristics of ferroptosis, such as levels of ROS, GSH, and MDA. As shown in **Figures 1F–H**, we found that quercetin exposure caused ROS accumulation, reduced GSH levels, and increased MDA levels in a dose-dependent manner. Additionally, we measured the expressions of ferroptosis-associated genes, and our results revealed that quercetin decreased the expression levels of NRF2, HO-1, GPX4, and FTH1, while it upregulated TFR1 in a dose-dependent manner (**Figures 1I–K**). Overall, our data suggest that ferroptosis plays a role in the quercetin-induced repression observed in OSCC cells.

### 3.2 Ferroptosis-dependent effect of quercetin against OSCC

**Figure 2:**
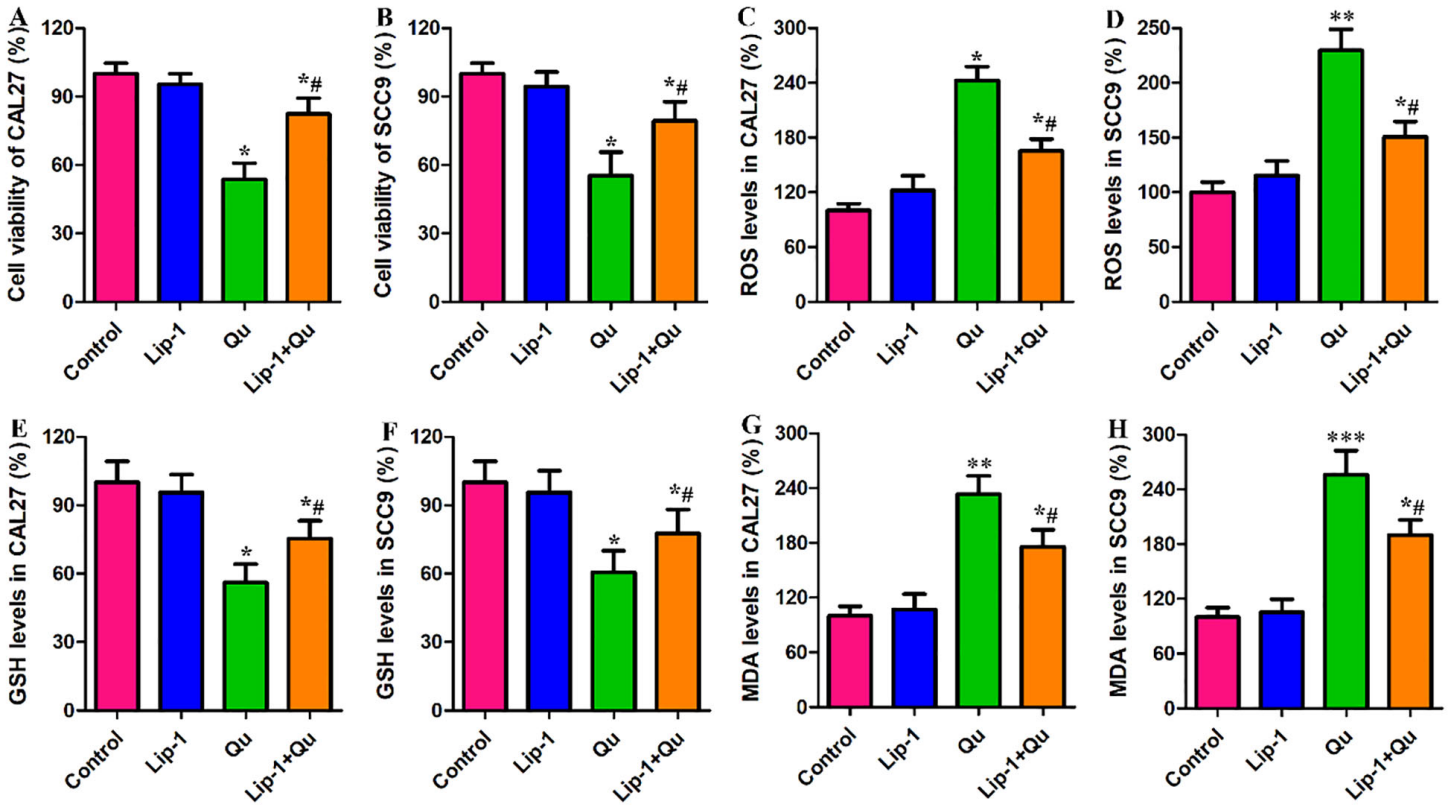
Quercetin-mediated cytotoxicity is dependent on ferroptosis. **(A, B)** Cell viability evaluation in CAL27 and SCC9 cells treated with or without Lip (1 μM) in the presence of quercetin for 24 h. **(C, D)** ROS, **(E, F)** GSH, **(G, H)** MDA levels in CAL27 and SCC9 cells treated with or without Lip (1 μM) in the presence of quercetin. Data were shown as the mean ± SD; the number of biological replicates was 3, ^*^P < 0.05, ^**^P < 0.01, compared with control group; ^#^*P* < 0.05, compared with Qu treatment group.

To further confirm that ferroptosis is the main form of cell death induced by quercetin in OSCC cells, we conducted rescue experiments with the ferroptosis inhibitor liproxstatin-1 (Lip-1). The CCK-8 assay showed that Lip-1 successfully reversed the quercetin-induced growth inhibition in CAL27 and SCC9 cells (**Figures 2A, B**). Moreover, Lip-1 also counteracted the increase in ROS levels in the cytoplasm, the decrease in GSH, and the rise in MDA observed in the quercetin-treated OSCC cells (**Figures 2C–H**). These results demonstrate that ferroptosis induction is the primary mechanism by which quercetin exerts its effects on OSCC.

### 3.3 Quercetin regulates GPX4-mediated ferroptosis by blocking LGR4/NF-κB pathway

**Figure 3:**
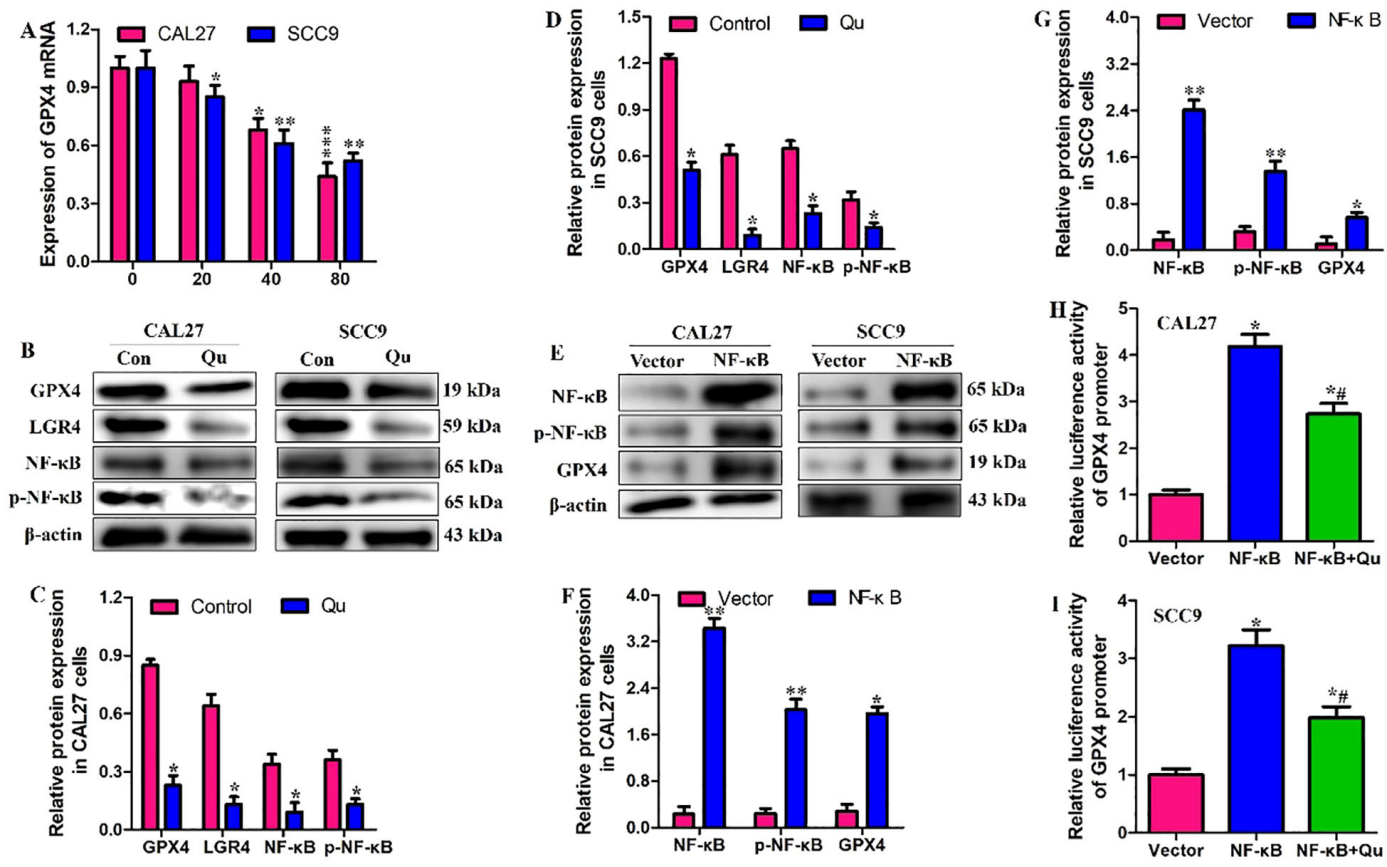
Quercetin inhibits LGR4/NF-κB/GPX4 axis to induce ferroptosis. **(A)** GPX4 mRNA expression in OSCC cells treated with different concentrations of quercetin for 24 h was measured by qRT-PCR. **(B–D)** The expression levels of GPX4, LGR4, NF-κB, and p- NF-κB in OSCC cells treated with or without quercetin (40 μM) were determined using western blotting. **(E–G)** Western blotting analysis of GPX4 expression induced by NF-κB overexpression in OSCC cells. **(H, I)** Luciferase reporter assays in CAL27 and SCC9 cells overexpressing NF-κB and GPX4 reporter plasmids treated with or without quercetin (40 μM). Data were shown as the mean ± SD; the number of biological replicates was 3, ^*^P < 0.05, ^**^P < 0.01, compared with control or vector group; ^#^*P* < 0.05, compared with NF-κB overexpression group.

Suppression of GPX4 activity causes lipid peroxide buildup, leading to ferroptosis [Ursini and Maiorino 2020 (13)]. Here, we further examined how quercetin affects GPX4 expression and the related molecular mechanisms. The results in **Figure 3A** show that quercetin reduced GPX4 mRNA levels in a dose-dependent way. Since NF-κB is a key transcription factor that controls GPX4 transcription, we investigated the effects of quercetin on the LGR4/NF-κB signaling pathway and found that quercetin notably decreased the levels of LGR4, NF-κB, and p-NF-κB. (**Figures 3B–D**). Subsequently, we overexpressed NF-κB in OSCC cell lines, which led to increased levels of GPX4 at both protein and mRNA levels (**Figures 3E–G**). To confirm the regulatory role of LGR4/NF-κB in GPX4, we cloned the GPX4 promoter into a dual-luciferase reporter construct and transfected it into OSCC cell lines. Notably, overexpression of NF-κB boosted the GPX4 promoter reporter activity, an effect that was countered by quercetin (**Figures 3H, I**). Overall, our findings suggest that quercetin modulates the LGR4/NF-κB/GPX4 axis, contributing to triggering ferroptosis.

### 3.4 LGR4-induced ferroptosis inhibition was rescued by quercetin and NF-κB inhibitor

**Figure 4:**
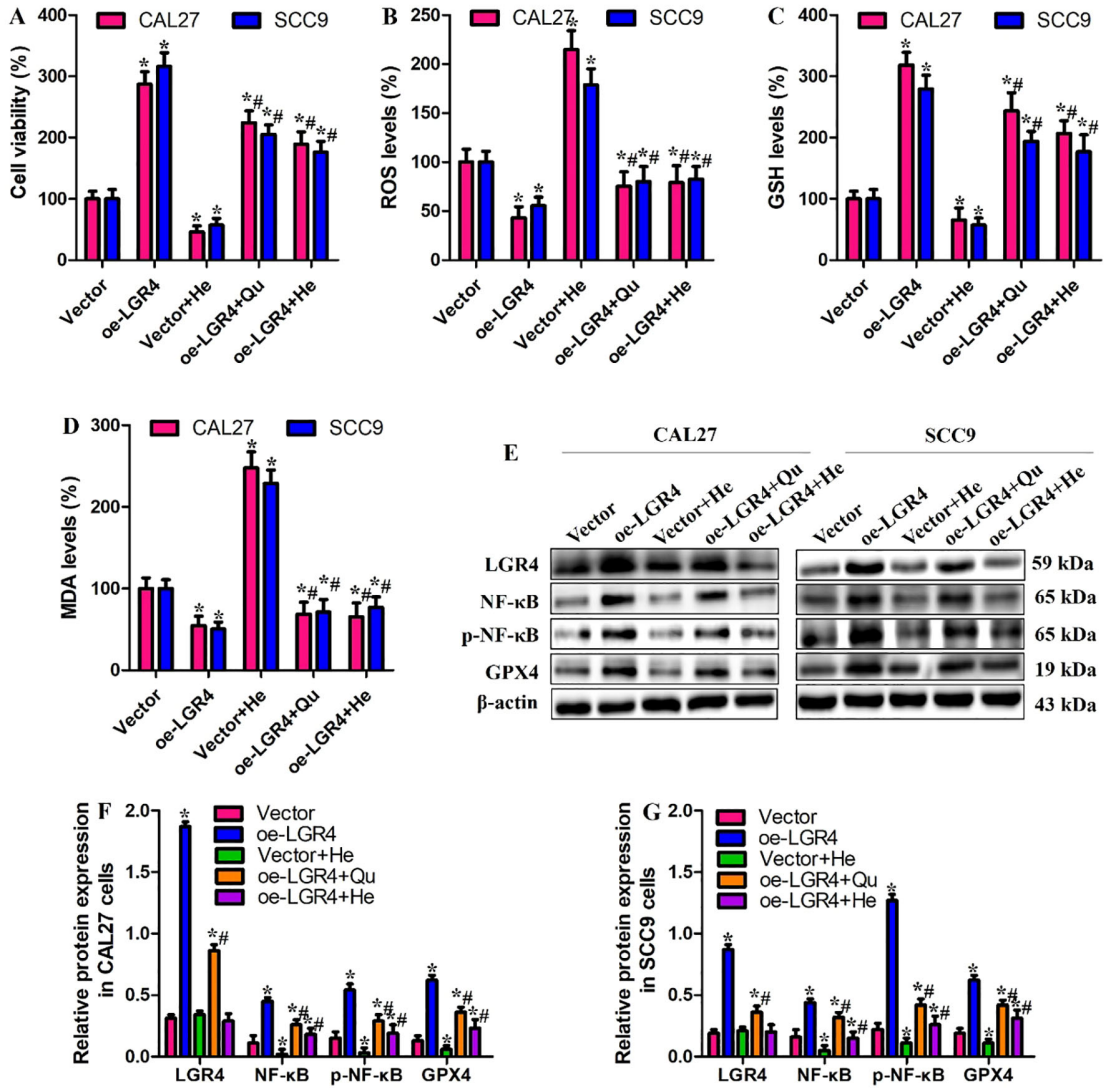
LGR4-induced ferroptosis suppression in OSCC cells was antagonized by quercetin and helenalin. **(A–D)** Cell viability, the levels of ROS, GSH, and MDA in OSCC cells transfected with vector or oe-LGR4 and treated with or without quercetin and helenalin (NF-κB inhibitor). **(E–G)** The expression levels of NF-κB, p- NF-κB, and GPX4 in cells transfected with vector or oe-LGR4 and treated with or without quercetin and helenalin. Data were shown as the mean ± SD; the number of biological replicates was 3, ^*^P < 0.05, compared with vector group; ^#^*P* < 0.05, compared with oe-LGR4 group.

To investigate the potential mechanisms of quercetin on OSCC, cells transfected with oe-LGR4 were exposed to quercetin (40 μM) or NF-κB inhibitor (helenalin, 2 μM). As demonstrated in **Figures 4A–D**, OSCC cells transfected with oe-LGR4 showed increased cell viability and GSH levels, along with decreased ROS and MDA. However, after exposure to quercetin, these effects were significantly counteracted, similar to the effects observed with helenalin treatment. Therefore, we hypothesized that NF-κB might be an important gene involved in OSCC caused by LGR4 overexpression. To explore the relationship between LGR4 and NF-κB, western blot analysis was performed. As shown in **Figures 4E–G**, cells overexpressing LGR4 exhibited increased levels of NF-κB, p-NF-κB, and GPX4. However, after treatment with quercetin, the expression of these proteins in cells transfected with oe-LGR4 was significantly reduced, similar to the effects observed in the oe-LGR4+ Helenalin group.

### 3.5 NF-κB-induced ferroptosis inhibition was antagonized by quercetin and the GPX4 inhibitor

**Figure 5:**
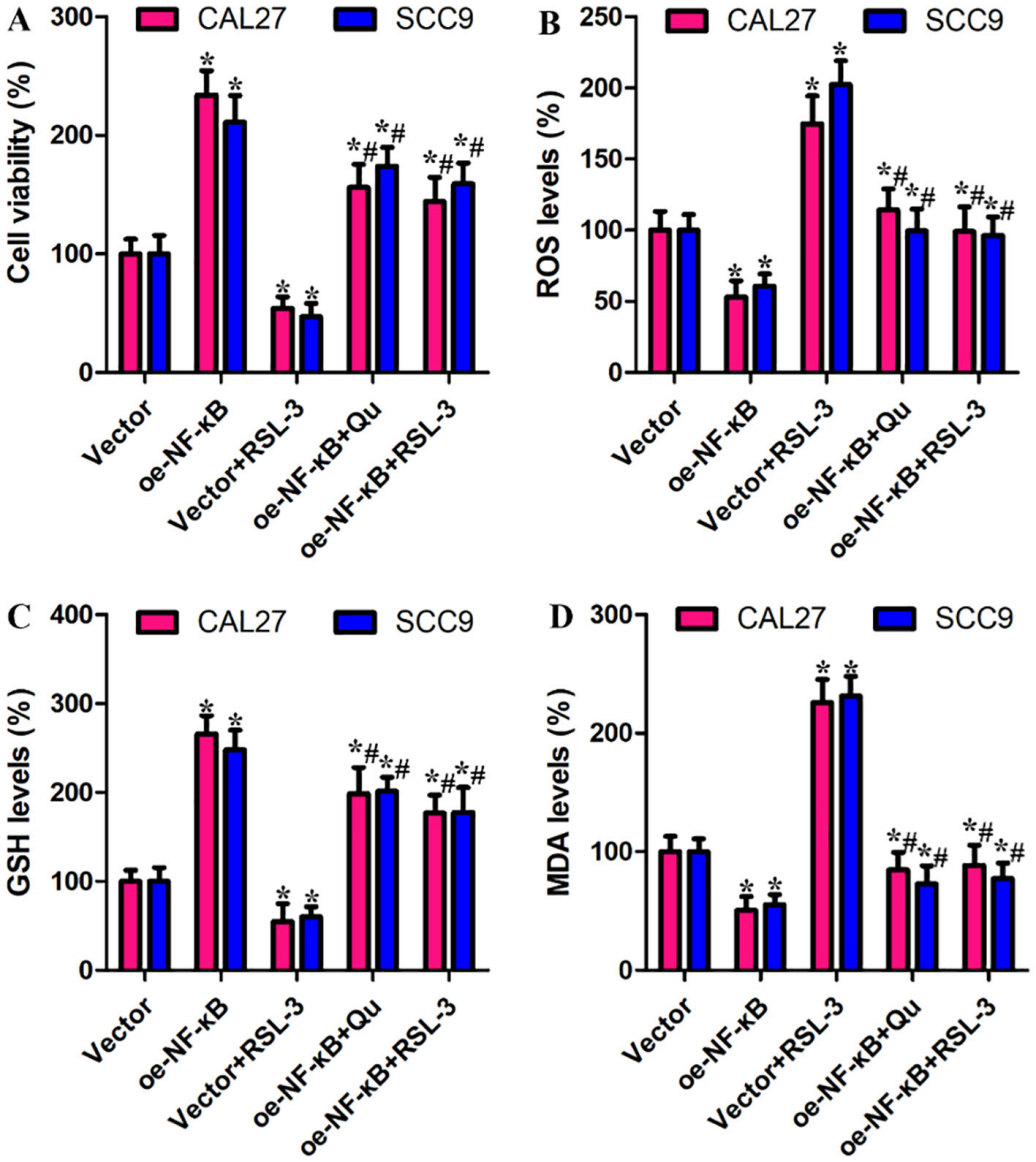
NF-κB-induced ferroptosis inhibition was reversed by quercetin and RSL-3. **(A)** Cell viability, **(B)** ROS, **(C)** GSH, **(D)** MDA levels in CAL27 and SCC9 cells transfected with vector or oe-NF-κB and treated with quercetin (40 μM) or RSL-3 (1.2 μM) for 24 h. Data were shown as the mean ± SD; the number of biological replicates was 3, ^*^P < 0.05, compared with vector group; ^#^*P* < 0.05, compared with oe-NF-κB group.

To explore the underlying role of NF-κB in quercetin-induced ferroptosis, OSCC cells transfected with oe-NF-κB were exposed to quercetin (40 μM) or GPX4 inhibitor (RSL-3, 1.2 μM). As shown in **Figures 5A–D**, OSCC cells transfected with oe-NF-κB exhibited increased cell viability and GSH levels, as well as decreased ROS and MDA levels. However, after exposure to quercetin, these effects were significantly counteracted, resembling those observed in the oe-NF-κB+RSL-3 group. These findings suggest that NF-κB is involved in the quercetin-induced ferroptosis in OSCC cells.

### 3.6 LGR4-induced ferroptosis repression was reversed by IKKβ deletion

**Figure 6:**
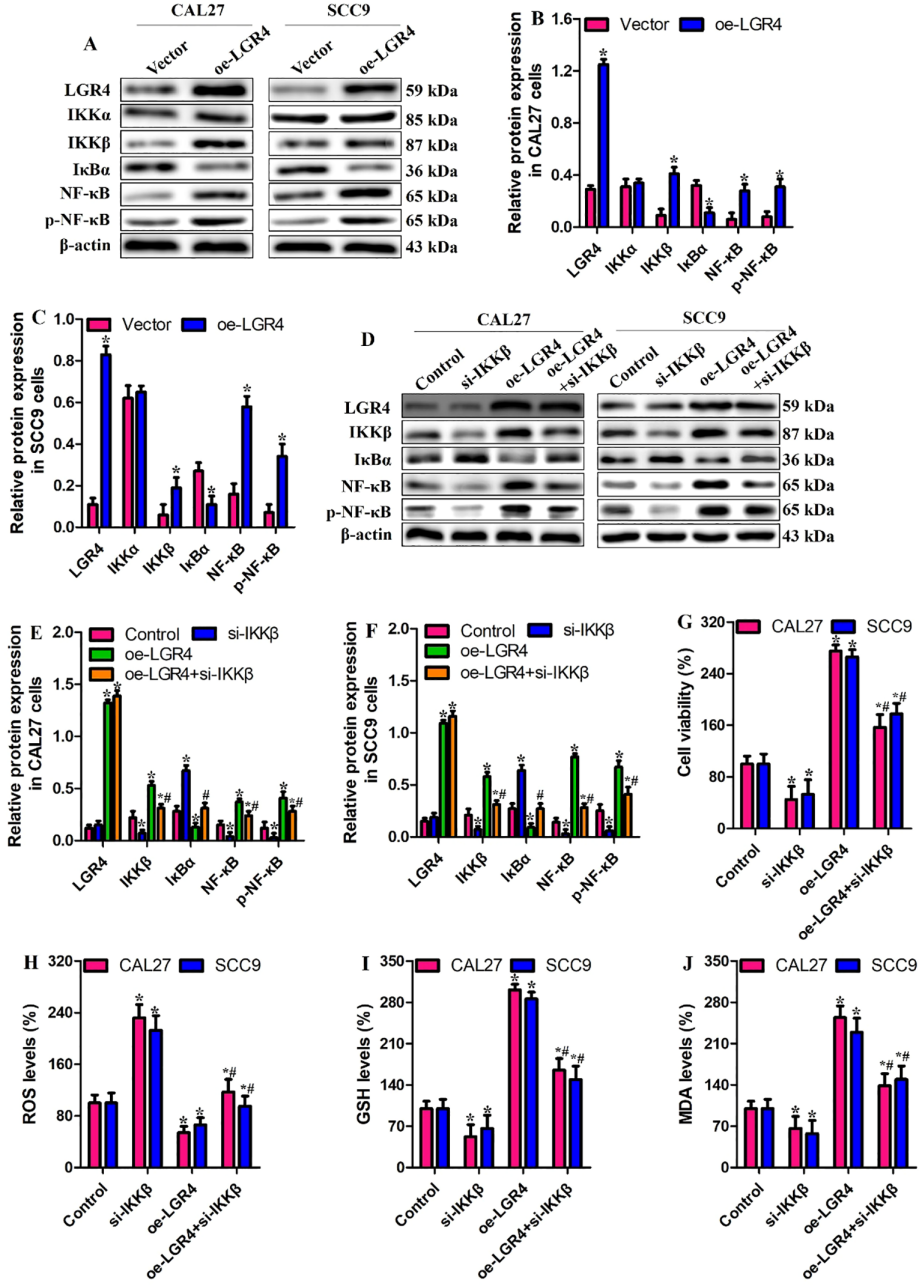
LGR4-induced ferroptosis inhibition by upregulating IKKβ. **(A–C)** Western blotting analysis of proteins in IKKβ/IκBα/NF-κB axis in cells transfected with vector or oe-LGR4. **(D–F)** Western blotting analysis of proteins in IKKβ/IκBα/NF-κB axis in cells transfected with si-IKKβ or oe-LGR4. **(G–J)** Cell viability, levels of ROS, GSH, and MDA were determined in cells transfected with si-IKKβ or oe-LGR4. Data were shown as the mean ± SD; the number of biological replicates was 3, ^*^P < 0.05, compared with control group; ^#^*P* < 0.05, compared with oe-LGR4 group.

It has been shown that IκBα, which forms a complex with NF-κB, can be ubiquitinated and targeted for degradation by IKKα and IKKβ [Fuentes et al., 2016 (14)]. As demonstrated in **Figures 6A–C**, LGR4 overexpression caused an increase in IKKβ, NF-κB, and p-NF-κB levels, while reducing IκBα, although it did not alter IKKα expression. To investigate the role of IKKβ in activating NF-κB, cells were transfected with si-IKKβ to reduce its expression. Deleting IKKβ led to increased IκBα expression and decreased NF-κB and p-NF-κB levels; additionally, the effects of LGR4 overexpression on the activation of the IKKβ/IκBα/NF-κB pathway were counteracted by IKKβ reduction (**Figures 6D–F**). Furthermore, similar results were observed in the levels of ferroptosis-related events in OSCC cells (**Figures 6G–J**).

## 4 Discussion

OSCC shows increased prevalence in men and older populations and has had a poor survival rate over the past decades (2). The most common treatments for OSCC are surgical resection, chemotherapy, and radiotherapy, but they can cause adverse effects such as mucositis, skin reactions, and dysphagia. Therefore, developing therapeutic agents to improve the prognosis and survival rates of OSCC is necessary. Here, in accordance with published reports (15, 16), we found that quercetin suppressed OSCC cell growth by inducing ferroptosis, with less toxicity in HOEC cells.

Cancer is marked by uncontrolled cell proliferation, so inhibiting cell growth has been regarded as an effective strategy for treating various cancers, including OSCC (17). Increasing evidence suggests that many plant-derived compounds are used in cancer treatment due to their role in regulating cell growth and death (18). Flavonols, which are widely found in plants, have been proposed as promising chemotherapeutic agents because of their low side effects (19). For instance, quercetin, one of the most common flavonols, has been shown to influence glycolysis and inhibit cell growth in OSCC (20). Studies have demonstrated that quercetin can suppress OSCC cell proliferation by modulating multiple signaling pathways, such as the microRNA-22/WNT1/beta-catenin axis (11), the SIRT3/AMPK/mTOR pathway (21), and the mTOR/S6K pathway (22). Our findings also indicate that quercetin induces ferroptosis in OSCC cells by repressing the LGR4/NF-κB/GPX4 axis. However, whether LGR4 is a direct molecular target of quercetin was not observed in the current study, and the upstream mechanisms through which quercetin transcriptionally regulates LGR4 expression have not been reported. Therefore, quercetin holds promise as a potential therapeutic agent for developing new treatments for anti-OSCC.

LGR4 is expressed in a wide range of tissues, and researchers have gradually understood its roles in cancer progression. On one hand, LGR4 was identified as an important gene that regulates oncogenesis, metastasis, and cancer stem cells in breast cancer. Increased LGR4 expression is regarded as a biomarker for predicting poor prognosis in breast cancer cases (22). On the other hand, LGR4 promotes the occurrence or development of cancers by regulating numerous signaling pathways, such as NF-κB transactivation. For example, LGR4 has been reported to enhance the inflammatory response in bovine endometrium damage, which is linked to NF-κB activation (23). Ordaz-Ramos et al. also provided evidence that LGR4 influences RANKL’s regulation of breast cancer stem cell properties through the NF-κB signaling pathway (24). Consistent with these reports, our current study shows that LGR4 is overexpressed in OSCC cells, and its overexpression suppresses ferroptosis. Therefore, we speculate that inhibiting LGR4 may be an effective strategy to improve OSCC outcomes by inactivating NF-κB.

To confirm our hypothesis, gene overexpression technology was used to control LGR4 expression and revealed that LGR4 overexpression promoted cell viability and reduced ferroptosis-associated events, as well as increased NF-κB phosphorylation in OSCC cells. Our results aligned with a published study indicating that NF-κB is highly expressed in multiple myeloma cells overexpressing LGR4 (9). Further observations demonstrated that LGR4 upregulation decreased ferroptosis in OSCC cells, though this trend was countered by both quercetin and an NF-κB inhibitor. These findings provide strong evidence that NF-κB plays a crucial role in LGR4’s regulation of ferroptosis in OSCC. Moreover, luciferase reporter assays showed that NF-κB can interact with the GPX4 promoter to enhance its transcription and trigger ferroptosis repression.

IκBα, which forms a complex with NF-κB, can be ubiquitinated for degradation by IKKα and IKKβ (14). Scholars have demonstrated that the suppression of NF-κB activation occurs during the period between the degradation and the re-synthesis of IκBα, which is a rapid IκBα-independent termination mechanism (25). Interestingly, we found that LGR4 can induce the upregulation of IKKβ expression and the downregulation of IκBα expression, suggesting that LGR4 promotes the upregulation of IKKβ to trigger IκBα ubiquitination and degradation, thereby enhancing NF-κB activation and GPX4 transcription, ultimately leading to ferroptosis in OSCC cells. However, it should be noted that our research has limitations, as we did not conduct animal studies or clinical trials to investigate the roles of quercetin in OSCC. We will further perform *in vivo* experiments to explore the effects of quercetin on ferroptosis and the LGR4/NF-κB/GPX4 axis in OSCC, thereby improving the completeness of our study.

## 5 Conclusion

In summary, we found that quercetin may induce ferroptosis in OSCC cells by specifically suppressing the LGR4/NF-κB signaling pathway, which reduces GPX4 transcription and ultimately causes ferroptosis. Therefore, quercetin-triggered ferroptosis may present a promising therapeutic target for the treatment of OSCC.

## Data Availability

The RNA-seq analysis project has been deposited at NCBI under BioProject ID (PRJNA1491029). The data presented in the study are deposited in the Figshare repository, DOI: 10.6084/m9.figshare.32791185.
